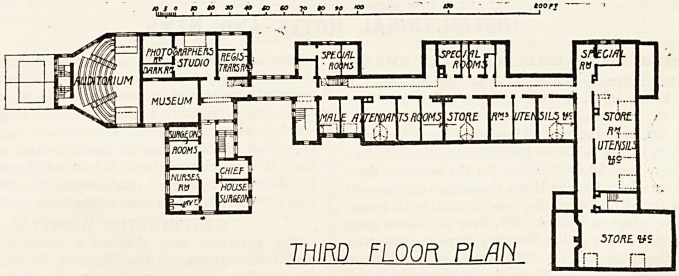# Some Modern Continental Hospitals

**Published:** 1909-09-11

**Authors:** 


### HOSPITAL ADMINISTRATION.

### CONSTRUCTION AND ECONOMICS.

### SOME MODERN CONTINENTAL HOSPITALS.

THE NEW SURGICAL CLINIC AT GREIFSWALD.

ALTHOUGH the small University centre of Greifswald cannot claim to possess an international fame, it can yet pride itself upon possessing one of the finest modern surgical hospitals. This is the new surgical clinic, which was completed in 1903 and has proved itself a thoroughly useful and efficient institution. Up to the date of its formal opening the surgical clinic had been located in the University Hospital, a building which was completed in 1856 and which was far from satisfactory in some respects. As it was recognised that the surgical division demanded a newer and better home, it was decided to give over 2,000 piles were driven into the ground, and on this a large platform was built on which the present buildings rest firmly. From the outside the new clinic presents a dignified and at the same time an attractive appearance, its red brick walls with cement facings and its tiled roof making it conspicuous, although there are no florid ornamentations and no attempt has been made to strive for great architectural beauty as in the newer Berlin hospitals. The large main front faces south, and the building consists really of two triple-storied blocks, one being the hospital itself, while the other serves for educational

FIRST FLOOR PLAN

THIRD FLOOR PLAN

this entire building to the medical department and to build a separate and entirely new block for the surgical side. All the plans and drawings were prepared in consultation with, and with the entire concurrence of, the chiefs of the surgical clinics—at first, in 1900, Professor Bier, and later on, when he accepted the call to Berlin, Professor Friedrich. Building operations were started in 1900 and completed in 1903. The clinic is unique in so far that it is probably the only German surgical hospital that is built on piles. This may at first sight justify a doubt as to the hygienic situation of the clinic, but we may point out that there are many hospitals which share this peculiarity, among others the fine institutions at Rotterdam, Amsterdam, and Venice. The architect cannot always claim the best site for his hospital; he has to use what is available. At Greifswald the only available plot for the new clinic was a piece of ground in which a firm foundation was only reached 21 feet below the surface. More than purposes. The following plans will make clear the arrangement of the grouping in the hospital block.

Fig. 1 (Plan A) shows the ground plan of the ground floor. This is the male division, and it will be seen that the demonstration rooms, to the left, are sharply separated from the wards proper by a corridor. The first floor ground plan is identical, with a few alterations on the west side, for above the polyclinic is the large operating theatre, with a small aseptic theatre and various extra rooms. In the hospital division there is no change. The wards are similar and the grouping is as on the ground floor. This first floor is used for female patients. The ground plan of the top floor is shown in fig. 2. On the second floor are found the auditorium of the large operating theatre, the photographic studio, and the quarters of the assistants and divisional surgeons, together with the rooms for the attendants, which are, however, situated on the east side. On the basement are the septic wards, with operating theatre, very full lavatory accommodation, and the usual extra rooms. On the second floor there is a large space reserved for a "sun-bath room."

A walk through the clinic shows the visitor that there is very little to criticise adversely. The institution is built on the corridor system, but the wards are so well lighted and airy that one hardly realises that they are separated on one side by a fairly wide corridor. Like all the modern German hospital wards they are scrupulously clean and comfortable, yet without any unnecessary furniture. The wards are floored with cement, covered by linoleum. In the operating rooms and lavatories they are of tiles. The ward walls are painted in a light colour; where necessary, as behind the basins, in the lavatories, and in the operating rooms, they are tiled to a distance of seven feet. Everywhere one notices the latest devices for preventing accumulation of dirt and dust. The window-frames and doors are rounded and close-fitting. What is specially striking is the excellent lavatory accommodation that has been provided. The cataract flush system is used and the waste-pipes are specially large and can be controlled at various points. In the polyclinic, as can be seen from the ground plan, the arrangement of the rooms is good, the male and female waiting rooms being separated by a corridor and lavatory. On the first floor a similar separation exists in the rooms attached to the operating theatre, there being separate male and female dressing, bandaging, and anæsthetising rooms. The apparatus rooms are found on the basement.

The clinic is heated by low-pressure steam pipes. The wards are heated and ventilated from below in winter, the heated air escaping through roof-ventilators. In summer natural ventilation suffices. In the operating rooms and theatre it is the only ventilation used. Water is laid on from the city mains; hot water is provided by a special system of pipes which, emerging from the basement boiler-house, radiate all over the building and make it possible for hot water, at an equal pressure, to be tapped off in any room. The wards and corridors are lighted by means of gas pendants; in the operating theatre electric light is found. The electricity used in the various departments in the clinic is entirely derived from the town mains. A special distillation apparatus is in use which provides distilled water for each ward and operating room—a great convenience in a surgical clinic. This hot water distillator is capable of yielding 30 litres of distilled water per hour, and was fitted up at a cost of £23. A full description and details of it will be found in the official account of the clinic published in the Klinisches Jahrbuch (18th volume) for 1908.

The operating theatre is large and airy, and is one of the best lit and most practical of the many new theatres after the Breslau type which have been built in Germany. It is directly connected with the female wards by a central corridor, and indirectly with the male wards on the floor below by means of the lifts, which are all of the automatic electric type. With it are connected the anæsthetising and preparing rooms. The auditorium is large and well designed, and as a lecture theatre the room serves admirably. The instrument cupboards are tiled inlets into the walls, closed by glass doors in steel frames.

Apart from the special rooms and the septic wards, the hospital can accommodate about 100 patients. The cost of the site was 76,100 marks; the total cost of construction 482,300 marks; of equipment, 94,[illegible]00. Without the cost of building site and equipment, the cost per bed works out at 4,820 marks : everything included, at approximately 6,500 marks.

## Figures and Tables

**Figure f1:**
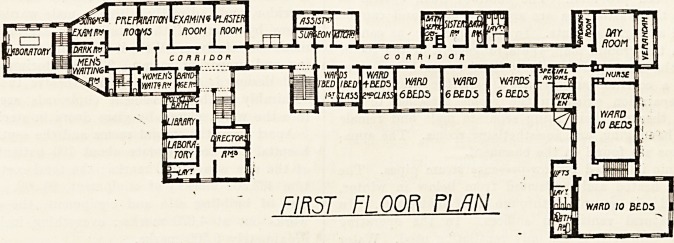


**Figure f2:**